# Lipoprotein A and the Association With Peripheral Arterial Disease: A Review of the Risk in Peripheral Arterial Disease

**DOI:** 10.31083/RCM41551

**Published:** 2025-11-13

**Authors:** Subrata Kar

**Affiliations:** ^1^Division of Cardiology, Virginia Commonwealth University Veterans Affairs Medical Center, Richmond, VA 23249, USA

**Keywords:** lipoprotein A, peripheral arterial disease, coronary artery disease

## Abstract

Peripheral arterial disease (PAD) is a global atherosclerotic disease which can lead to acute limb ischemia, chronic limb-threatening ischemia, and limb amputation. It has similar risk factors to coronary artery disease (CAD). Elevated lipoprotein A (Lp[a]) is associated with CAD, myocardial infarction, and PAD. Patients with PAD can have CAD and polyvascular disease. An extensive PubMed and Cochrane library search was performed in April 2025 using the words “Lipoprotein A and PAD”, “Elevated lipoprotein A and PAD”, and “High Lipoprotein A and PAD” to obtain relevant English articles for this systematic review. An elevated Lp(a) may enhance the risk of PAD. Elevated Lp(a) can amplify the risk of CAD, PAD, and polyvascular disease. It may portend worse outcomes in patients with CAD and PAD. It can increase the risk of acute limb ischemia, coronary revascularization, peripheral revascularization, cardiovascular death, and all-cause mortality. Hence, elevated Lp(a) may serve as a risk factor for patients with CAD who could potentially develop PAD. No currently approved medical therapy aimed at Lp(a) reduction exists; only lipoprotein apheresis is approved to lower Lp(a) levels in these patients. This systematic review discusses the role of an elevated Lp(a) in PAD, clinical research in PAD with elevated Lp(a), and the current treatment for PAD and elevated Lp(a).

## 1. Introduction

Lipoprotein A (Lp[a]) is associated with an increased risk of coronary artery disease (CAD) [1]. Lp(a) is also associated with myocardial infarction (MI), stroke, and aortic stenosis [2]. Lp(a) acts as an additional risk factor for CAD in addition to the traditional risk factors of diabetes, hypertension, hyperlipidemia, chronic kidney disease, obesity, and tobacco use. Lp(a) is also associated with an increased risk of premature CAD along with more extensive CAD in South Asians [1]. In patients with early onset CAD or extensive CAD, elevated Lp(a) should be considered a risk factor. The 2019 American College of Cardiology/American Heart Association guideline on the primary prevention of cardiovascular disease lists elevated Lp(a) [≥50 mg/dL or ≥125 nmol/L] and having a South Asian ancestry as a risk-enhancing factor [3].

Moreover, Lp(a) is also linked with an enhanced risk of peripheral arterial disease (PAD) [4]. PAD can increase the risk for adverse vascular events such as decreased quality of life from intermittent claudication, lower extremity ulcers, limb ischemia, limb amputation, and mortality. In patients with PAD, CAD may co-exist along with other vascular diseases leading to polyvascular disease, underscoring the importance of PAD evaluation and diagnosis [5, 6]. In addition, understanding and optimizing medical therapy for PAD is integral to reducing the risk of such adverse events. In this systematic review, the role of elevated Lp(a) in PAD will be discussed along with the clinical studies and contemporary management options.

## 2. Methods

**Fig. 1.**
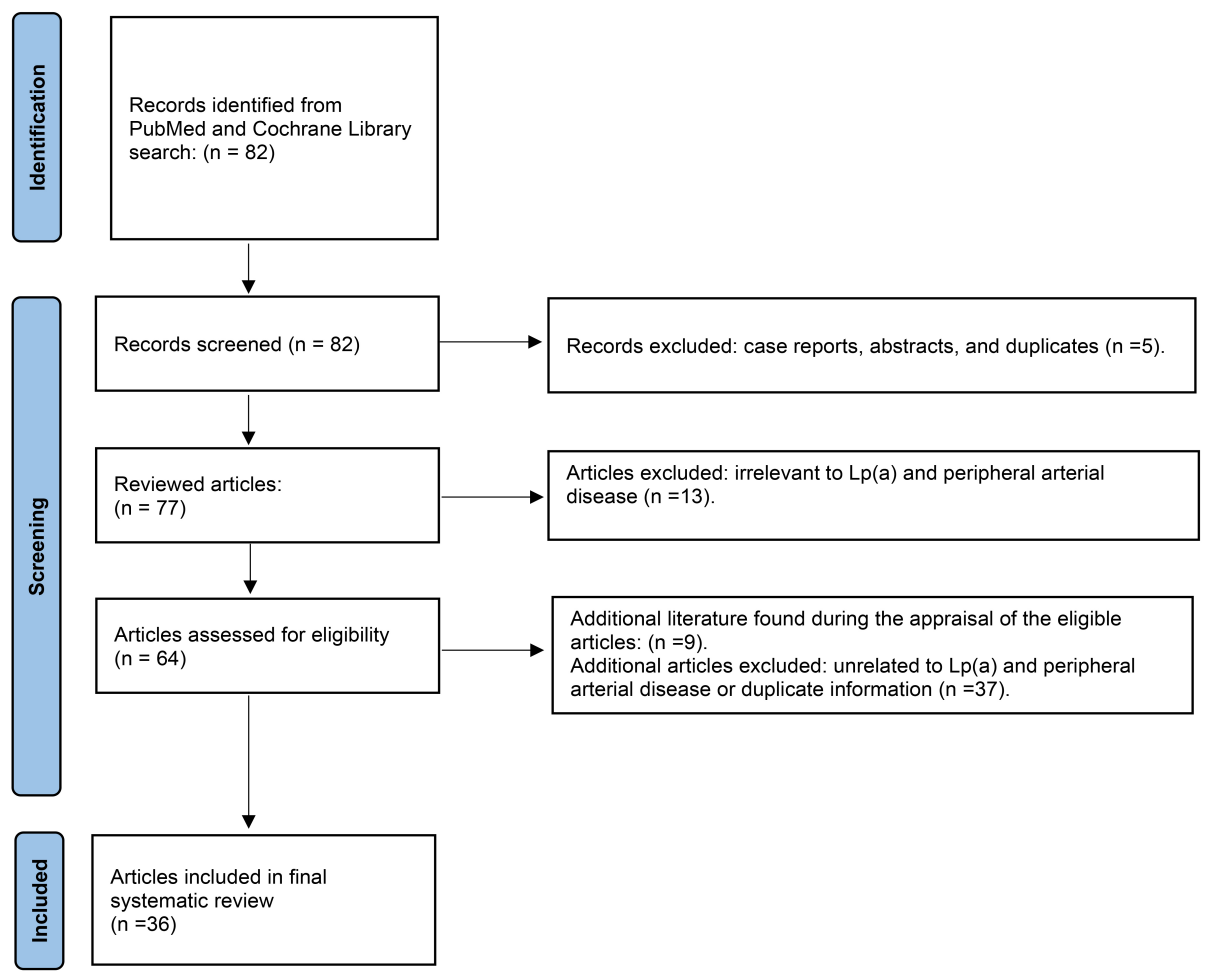
**Search method using preferred reporting items for systematic reviews and meta-analyses (PRISMA) for the systematic review of clinical studies with elevated lipoprotein A in peripheral arterial disease**.

An extensive PubMed and Cochrane library search using the Preferred Reporting Items for Systematic reviews and Meta-Analyses (PRISMA) method was performed in April 2025 using the words, “Lipoprotein A and PAD”, “Elevated lipoprotein A and PAD”, and “High Lipoprotein A and PAD” to obtain relevant English articles for this systematic review (Fig. 1). A total of 82 articles was found, of which, 64 involved PAD and Lp(a). Articles were excluded if they were duplicates, case reports, abstracts, and/or unrelated to the search criteria. Clinical trials, observational studies, meta-analyses, reviews, and editorials were included. Only studies performed in adults and published in English were included. No date restrictions were applied for article exclusion. Additional articles were found from the selected manuscripts to comprise the final reference list (n = 36).

## 3. PAD

PAD affects over 200 million people globally [6]. In the United States alone, an estimated 12 million people may be affected by PAD [7]. However, 50% of individuals with PAD may be asymptomatic [8]. Thus, the global population truly affected by PAD may be underestimated. PAD risk factors are similar to CAD which includes age (≥75), diabetes, hypertension, hyperlipidemia, obesity, chronic kidney disease, end stage renal disease, polyvascular disease (coexisting atherosclerosis in other vascular territories-coronary, peripheral, or cerebrovascular) and tobacco use [9]. PAD is associated with cardiovascular disease and limb amputations [8]. PAD patients can progress to chronic limb threatening ischemia/critical limb ischemia (CLI) (estimated incidence of 11–20%) with a 1 year mortality rate of 25–35% and a 1 year amputation rate of 30% [9]. It can increase the risk of CAD by 45% and cerebrovascular disease by 35% [10]. It can increase the risk of Major Adverse Cardiac Events (MACE) with a greater risk of cardiovascular death, MI, and stroke [5]. Therefore, diagnosing and adequately managing PAD can improve quality of life, reduce limb amputation, and improve overall cardiovascular health.

## 4. Lp(a) Biology and Mechanisms

Lp(a) consists of a low-density lipoprotein (LDL) particle with an oxidized phospholipid which is attached to apolipoprotein B100 and covalently bound to apolipoprotein A [1]. It is primarily genetically derived and non-modifiable with statins [1]. Lp(a) can be 80–90% genetically predetermined and has been linked to MI, stroke and cardiovascular mortality [2]. Lp(a) is relatively unchanged during an individual’s lifetime; thus, repeat measurements are unnecessary [1].

Lp(a) can stimulate the development of atherosclerosis, smooth muscle cell calcification in blood vessels, and inflammation. It can also promote thrombogenesis through its homology with plasminogen [6]. The oxidized phospholipid in Lp(a) can induce stress and inflammation through foam cell formation via the activation of monocytes and endothelial cells [6]. Oxidized phospholipids enter cells and arterial walls, inducing inflammation leading to lipid deposition in arteries, resulting in atherosclerosis. This enhances the risk of atherosclerotic CAD, PAD, and polyvascular disease. In the circulation, 85% of oxidized phospholipids are bound to Lp(a) [6]. Moreover, apolipoprotein A which is also attached to Lp(a) is another molecule which promotes the adverse effects of Lp(a). Apolipoprotein A competes with plasminogen to bind to endothelial cells and fibrin due to its homology with plasminogen [6]. Such an interaction can induce thrombosis by reducing the activation of plasminogen to plasmin which is necessary for fibrinolysis. Lp(a) also causes increased platelet aggregation via CD36 and protease-activated receptor 1 (PAR 1) [6].

## 5. Lipoprotein A and PAD risk

Elevated Lp(a) can be linked to a heightened risk of PAD and polyvascular disease.

The mechanisms by which Lp(a) induces atherogenesis can affect both the coronary and peripheral vessels. It can incite worse outcomes in patients with elevated Lp(a) and high LDL. It may promote more severe PAD and poor outcomes in PAD. Thus, elevated Lp(a) can induce CAD, PAD, and polyvascular disease. Complications from PAD may include major adverse limb events (MALE). MALE includes complications from PAD such as acute limb ischemia, limb amputation, critical limb threating ischemia, progression from intermittent claudication (IM) to critical limb threating ischemia, or repeat peripheral intervention for limb ischemia. Limb amputation can occur in patients with PAD as the disease progresses. Amputations may include toe amputation, transmetatarsal amputation, below the knee amputation, or above the knee amputation. Revascularization includes intervention for patients with PAD such as balloon angioplasty, drug-coated balloon angioplasty, or peripheral stent implantation. Furthermore, Lp(a) concentrations can be affected by liver disease, renal failure, and sex hormones. For example, estrogen, androgen esters, and estrogen-progestin combinations can lower Lp(a) [6]. Consequently, postmenopausal women can have increased Lp(a) levels. In addition, it can be increased in kidney disease, growth hormone therapy, pregnancy, and hypothyroidism. Conversely, it can be decreased in hyperthyroidism, liver disease, severe acute phase conditions, and by postmenopausal hormone replacement therapy [11].

## 6. Clinical Relevance and Treatment Options for Elevated Lp(a)

### 6.1 Clinical Studies of PAD and Lp(a) 

In the Copenhagen General Population Study (prospective cohort study) of 108,146 patients, 2450 patients had PAD [4]. In these PAD patients, elevated Lp(a) was associated with an increased risk of PAD and abdominal aortic aneurysm (AAA). It also increased the risk of MALE. Patients with Lp(a) levels ≥99th (≥143 mg/dL, ≥307 nmol/L) vs. <50th percentile (≤9 mg/dL, ≤17 nmol/L) had a multivariable-adjusted hazard ratio (HR) of 2.99 (95% Confidence Interval [CI]: 2.09–4.30) for PAD. The incidence rate ratio for MALE was 3.04 (95% CI: 1.55–5.98). In male smokers (aged 70–79 years) with Lp(a) <50th and ≥99th percentile, absolute 10-year risk of PAD was 11% and 29% and in female smokers was 8% and 21%, respectively. Thus, higher Lp(a) levels in the general population increased the risk of PAD, AAA, and MALE by 2–3-fold. Table 1 (Ref. [4, 12, 13, 14, 15]) lists the clinical trials of elevated Lp(a) and PAD.

**Table 1.** **Clinical Studies of Elevated lipoprotein A and the Association with PAD**.

Study (type)	Patients (n)	Outcomes	Results
Copenhagen General Population Study (prospective cohort study) [4]	108,146 (PAD, n = 2450)	PAD, AAA, MALE	Elevated Lp(a) > risk of PAD, AAA, MALE
InCHIANTI (prospective study) [12]	1002	PAD incidence	Elevated Lp(a) [≥32.9 mg/dL)] > PAD
EPIC-Norfolk (prospective study) [13]	18,720 (PAD, n = 596)	PAD, CAD, ischemic stroke	Lp(a) = adverse PAD and CAD outcomes
Small *et al*. (population cohort study) [14]	3 populations (United Kingdom Biobank, n = 357,220; other 2 populations, n = 34,020)	Risk of MACE (composite of cardiac death, MI, ischemic stroke), individual MACE components, PAD	Elevated Lp(a) > MACE, MI, PAD
Sakata *et al*. (prospective study) [15]	1104	Limb events, cerebrovascular or cardiac death, all-cause death, MACE	High Lp(a) [≥21 mg/dL)] > limb events, cardiac death, all-cause death, MACE, critical limb ischemia

PAD, Peripheral Arterial Disease; AAA, Abdominal Aortic Aneurysm; MALE, Major Adverse Limb Events; Lp(a), Lipoprotein A; CAD, Coronary Artery Disease; MACE, Major Adverse Cardiac Events; MI, Myocardial Infarction.

In 242 Japanese patients with CLI (n = 42) or IM (n = 200), Lp(a) and high sensitivity troponin was greater in the CLI group than the IM group (45.9 ± 23.3 mg/dL vs. 26.2 ± 27.7, *p* = 0.0002; 0.152 ± 0.186 ng/mL vs. 0.046 ± 0.091, *p* < 0.0001) [16].

In the InCHIANTI prospective study [12] of 1002 Italian patients (ages 60–96), Lp(a) was an independent predictor of PAD. Moreover, highest Lp(a) quartiles (≥32.9 mg/dL) had an increased likelihood for PAD compared with the lowest quartile (odds ratio, OR = 1.83, 95% CI = 1.01–3.33).

The EPIC-Norfolk prospective population study [13] of 18,720 patients (age 39–79 years old) in Norfolk, United Kingdom (CAD, n = 2365; ischemic stroke, n = 284; PAD, n = 596) revealed that Lp(a) was associated with adverse PAD and CAD outcomes, but not ischemic stroke. LDL did not modify these risks.

In the ODDYSSEY OUTCOMES trial [17], the risk of PAD in patients with recent acute coronary syndrome was associated with the levels of Lp(a), which was diminished by the proprotein convertase subtilisin/kexin type 9 (PCSK9) inhibitor, alirocumab. Similarly, the FOURIER trial (Further Cardiovascular Outcomes Research With PCSK9 Inhibition in Subjects with Elevated Risk) [18] of 27,564 patients with atherosclerotic disease showed that evolocumab (PCSK9 inhibitor) reduced MALE (acute limb ischemia, major amputation, or urgent ischemia induced peripheral revascularization) in PAD patients (n = 3642; no past MI or stroke, n = 1505; HR: 0.58, 95% CI 0.38–0.88; *p* = 0.0093). It also reduced the composite of cardiovascular death, MI, stroke, hospital admission for unstable angina or coronary revascularization (HR: 0.79, 95% CI 0.66–0.94, *p* = 0.0098).

A cohort study by Small *et al*. [14] of 3 populations (United Kingdom Biobank without CAD; FOURIER TIMI 59 trial; SAVOR-TIMI 53 trial with CAD-Saxagliptin Assessment of Vascular Outcomes Recorded in Patients with Diabetes Mellitus trial) showed that higher levels of Lp(a) was associated with an increased risk of MI, MACE, and PAD, irrespective of their high sensitivity-C reactive protein (hs-CRP) levels. Patients with higher Lp(a) had an increased cardiovascular risk for MACE (regardless of hs-CRP), MI, ischemic stroke, and PAD (hs-CRP ≥2 mg/L: HR per 50-nmol/L higher Lp(a), 1.05; 95% CI 1.04–1.07; *p* < 0.001; hs-CRP <2 mg/L: HR: 1.05; 95% CI 1.04–1.07; *p* < 0.001). In the aggregated data from the FOURIER and SAVOR trials, higher baseline Lp(a) was associated with an increased cardiovascular risk for MACE, MI, and PAD (hs-CRP ≥2 mg/L: HR per 50-nmol/L higher Lp(a), 1.02; 95% CI 1.00–1.05; *p* = 0.04; hs-CRP <2 mg/L: HR: 1.05; 95% CI 1.02–1.08; *p* < 0.001).

In the post hoc secondary analysis of the International Examining Use of Ticagrelor in Peripheral Arterial Disease (EUCLID) trial [19] of 13,885 patients with PAD (4 geographical regions-Central/South America, Europe, Asia, North America), monotherapy with ticagrelor and clopidogrel was compared, which showed equal effectiveness in symptomatic PAD [18]. The risk of MACE and lower extremity revascularization was greater in patients with polyvascular disease (CAD, PAD, and cerebrovascular disease) [19]. The outcomes of patients with CLI (5%) from the EUCLID trial [19] showed a significantly higher rate of cardiovascular mortality and morbidity compared to those sans CLI.

In a retrospective Australian study of 1472 patients, Lp(a) ≥30 mg/dL was associated with a higher requirement for PAD surgery (HR: 1.20, 95% CI 1.02–1.41) [20]. It was also associated with greater lower limb peripheral revascularization (HR: 1.33, 95% CI 1.06–1.66) without an increased risk of MACE or all-cause mortality. Moreover, Lp(a) ≥50 mg/dL was linked with an enhanced risk of lower limb peripheral revascularization without other adverse outcomes [20].

Sakata *et al*. [15] conducted an observational prospective study of 1104 patients (median follow-up of 68 months), and found that elevated Lp(a) ≥21 mg/dL worsened leg events (new peripheral lesion, repeat peripheral revascularization, or major amputation), cardiovascular-related death, all-cause death, and MACE (*p* < 0.05). Elevated Lp(a) was associated with an increased C-reactive protein (CRP), d-dimer, and LDL with a lower estimated glomerular filtration rate. Moreover, high Lp(a) was associated with a greater incidence of CLI.

Yanaka *et al*. [21] performed a retrospective study of 109 patients (mean follow-up of 28 months) who underwent endovascular intervention for femoropopliteal lesions with high Lp(a) ≥30 mg/dL (n = 31) or low <30 mg/dL (n = 78). The primary patency rates of low vs. high Lp(a) at 1 year were 83% and 76% and at 2 years were 75% and 58%, respectively (*p* = 0.02). High Lp(a) ≥30 mg/dL (HR: 2.44, 95% CI 1.10–5.44, *p* = 0.03) was an independent predictor of loss of primary patency of revascularization. Correspondingly, a prospective single center observational study of 1169 Japanese patients (median follow-up of 1.7 years) who underwent endovascular intervention for symptomatic PAD (33.4% with chronic limb threatening ischemia) showed that elevated Lp(a) ≥30 mg/dL was independently associated with MACE (all-cause death, MI, and stroke) and MALE (repeat limb revascularization and amputation) regardless of the LDL level and statin administration [22]. The cumulative incidence rate of MACE (48.1% vs. 27.3%) and MALE (67.9% vs. 27.2%) was higher with high Lp(a) ≥30 mg/dL [*p* < 0.001]. The adjusted HR for MACE was 1.93 (95% CI: 1.44–2.59; *p* < 0.001) and MALE was 4.15 (95% CI: 3.14–5.50; *p* = 0.001).

A systematic review of 15 studies (n = 493,650) revealed that elevated Lp(a) was associated with a greater risk of claudication (relative risk: 1.20), PAD progression (HR: 1.41), restenosis (HR: 6.10), death and hospitalization related to PAD (HR: 1.37), limb amputation (HR: 22.75), and lower limb revascularization (HR: 1.29 and 2.90) [23]. Elevated Lp(a) was also associated with a higher risk of combined PAD outcomes (HR range of 1.14 to 2.80). The 15 study analyses concluded that elevated Lp(a) was associated with a higher risk of claudication (32%), PAD progression (41%), PAD restenosis (approximately sixfold), death and hospitalization related to PAD (37%), limb amputation (about 22-fold), and lower limb revascularization (about threefold) [23]. Elevated Lp(a) was associated with a higher risk of combined PAD outcomes (range of 14%–280%), which persisted even after adjustment for the traditional risk factors [23].

### 6.2 Medical Treatment for PAD

Medical therapy is recommended for PAD to reduce the risk of progression into chronic limb-threatening ischemia, acute limb ischemia, limb amputation and/or death. Medical therapy consists of single antiplatelet therapy with aspirin [9]. Clopidogrel can be used as an alternative [9]. In the PEGASUS-TIMI 54 (Prevention of Cardiovascular Events In Patients with Prior Heart Attack Using Ticagrelor Compared to Placebo on a Background of Aspirin-Thrombolysis in Myocardial Infarction 54) [24] randomized controlled trial of 21,162 patients with prior MI (1–3 years) treated with aspirin plus ticagrelor 90 mg twice daily, ticagrelor 60 mg twice daily, or placebo, patients with PAD (n = 1143; 5%) had an increased ischemic risk. PAD patients (placebo arm, n = 404) had a greater likelihood of MACE at 3 years than without PAD (n = 6663; 19.3% vs 8.4%; *p* <0.001). PAD patients also had a greater rate of acute limb ischemia (1.0% vs. 0.1%) and peripheral revascularization (9.15% vs. 0.46%). Patients treated with ticagrelor (pooled doses) had a reduced risk of MALE-composite of acute limb ischemia and peripheral revascularization for ischemia (HR: 0.65, 95% CI 0.44 to 0.95; *p* = 0.026). Ticagrelor also lowered MACE (absolute risk reduction of 4.1%).

In symptomatic PAD, aspirin 81 mg daily combined with rivaroxaban 2.5 mg twice daily is recommended to reduce the incidence of MACE or MALE [9]. In patients who have undergone peripheral endovascular or surgical intervention, combined therapy with aspirin and rivaroxaban is also advised to reduce MACE or MALE [9]. The COMPASS (Cardiovascular Outcomes for People Using Anticoagulation Strategies) trial [25] of PAD patients (n = 6391) showed a significant reduction of MALE (43%, *p* = 0.01), total vascular amputations (58%, *p* = 0.01), peripheral vascular interventions (24%, *p* = 0.03), and all peripheral vascular outcomes (24%, *p* = 0.02) using rivaroxaban 2.5 mg twice daily and aspirin.

After endovascular intervention, dual antiplatelet therapy with aspirin 81 mg daily and clopidogrel 75 mg daily is reasonable for 1–6 months [9]. If patients are receiving anticoagulation for another indication, then adding a single antiplatelet is reasonable if they are not at a high bleeding risk [9]. The nuances of such medical therapy should be discussed with the patient’s interventional cardiologist, general cardiologist, or vascular surgeon to tailor the optimal individualized therapy.

### 6.3 Treatment Options for an Elevated Lp(a)

No current medical therapy targeting Lp(a) exists which is approved by the Food and Drug Administration (FDA). Clinical trials evaluating small interfering ribonucleic acids (siRNA) and antisense oligonucleotides (ASO) for targeted Lp(a) reduction are currently ongoing. In the ORION-1 [26] randomized controlled trial (phase 2) of 501 patients with CAD or CAD risk equivalents with high LDL (treated with maximally tolerated statins), inclisiran (synthetic siRNA targeting PCSK9 messenger RNA synthesis) showed a reduction in LDL, apolipoprotein B, non-high density lipoprotein (HDL) cholesterol, and Lp(a) with an increase in HDL. The ORION-10 (n = 1561, phase 3) and ORION-11 trials (n = 1617, phase 3) reported a 21.9% and 18.6% median reduction of Lp(a) using inclisiran (treated with zetia and maximally tolerated statins), respectively [27]. In a phase 2b randomized, double blind, placebo-controlled dose-ranging trial of 286 patients with CAD and Lp(a) ≥60 mg/dL (≥150 nmol/L), pelacarsen (hepatocyte-directed ASO) significantly lowered Lp(a), oxidized phospholipids on apolipoprotein (a) and apolipoprotein B-100, and mildly reduced LDL [28].

The PCSK9 inhibitor, alirocumab, reduced LDL and Lp(a) in the ODDYSSEY OUTCOMES trial [17]. Likewise, evolocumab (PCSK9 inhibitor) reduced Lp(a) at 48 weeks by a median of 26.9% (interquartile range of 6.2%–46.7%) along with LDL in the FOURIER trial [29]. In a network meta-analysis of PCSK9 inhibitors, the best treatment producing a reduction in Lp(a) of up to 25.1% was a biweekly dose of evolocumab 140 mg or alirocumab 150 mg [30].

Alternatively, lipoprotein apheresis approved by the FDA for elevated Lp(a) can reduce Lp(a) levels, which involves removing LDL and Lp(a) from the serum. If lifestyle and pharmacologic therapy are unable to decrease elevated Lp(a), Lp(a) apheresis can remove it from the plasma, which is usually well tolerated. Indications for apheresis include patients with homozygous or heterozygous Familial Hypercholesterolemia (FH) and LDL cholesterol levels >300 mg/dL, heterozygous FH and high CAD risk with an LDL cholesterol >200 mg/dL, heterozygous FH and CAD or diabetes with an LDL cholesterol >160 mg/dL, or progressive CAD and Lp(a) concentrations >60 mg/dL [31]. Lipoproteins can be extracted from the plasma by heparin precipitation, adsorption (binding to polyacrylate anions or dextran sulfate), or filtration (filters remove lipoproteins based on size) with an estimated reduction of 50–75% [29]. Triglycerides may be reduced by 50%. Nevertheless, after 8–13 days the LDL cholesterol and Lp(a) level may return to baseline in some patients [31]. The triglyceride and HDL cholesterol return to baseline within 24 hours [31].

Lp(a) apheresis can reduce major coronary events in CAD [32]. It can also reduce rates of MALE, limb amputation, and induce symptom improvement in PAD [32]. In a longitudinal, multicenter, cohort study of 120 patients, Lp(a) apheresis added to maximally tolerated statins lowered Lp(a) levels (*p* < 0.0001) with a reduction in mean annual MACE per patient of 1.056 vs. 0.144 (*p* < 0.0001) [33]. In a prospective observational multicenter study of 170 patients, mean annual rates of MACE declined with Lp(a) apheresis from 0.41 (without apheresis) to 0.09 (2 years of apheresis (*p* < 0.0001) [34]. Moreover, the event rates of all the vascular beds with apheresis treatment decreased from 0.61 to 0.16 (*p* < 0.0001). In a study of 10 patients with elevated Lp(a) and PAD, apheresis reduced PAD revascularization [35]. In the 12 months preceding apheresis, 35 peripheral procedures occurred in 120 patient-months. During the 2-year follow-up period with apheresis, only 2 procedures occurred in 229 patient-months (*p* < 0.001) [35]. Thus, Lp(a) is an important marker for PAD and reduction is potentially beneficial. Nonetheless, Lp(a) apheresis is expensive and can require additional treatment, so it is not easily applicable in clinical practice. Lp(a) apheresis is also not readily available at every medical center.

## 7. Discussion 

PAD is a global health concern since many patients are asymptomatic, and symptomatic patients may have atherosclerotic disease in other vascular beds (polyvascular disease) with resultant acute limb ischemia, chronic limb-threatening ischemia/CLI, limb amputation, and death. PAD patients have an increased risk of CAD, MI, stroke, acute limb ischemia, coronary revascularization, peripheral revascularization, cardiovascular death, and all-cause mortality [24].

Elevated Lp(a) can serve as a risk factor for CAD, premature CAD, and multivessel CAD [1]. South Asians have elevated Lp(a) so they can develop early CAD [1]. Indications for Lp(a) measurement include a family history of premature CAD [3]. Elevated Lp(a) can increase the risk of PAD and engender a heightened propensity for peripheral revascularization. Various clinical studies have shown that an elevated Lp(a) can increase the risk for PAD or worsen outcomes in PAD, which can be reduced with Lp(a) apheresis. Furthermore, patients who undergo successful peripheral endovascular revascularization with an elevated Lp(a) can experience more recurrences and PAD hospitalizations.

Numerous studies have different Lp(a) values indicating worse outcomes in PAD. However, no consistent values were found. Thus, it is difficult to ascertain a specific value at which the risk of PAD or polyvascular disease may be increased. Moreover, some studies use different measurement values such as mg/dL or nmol/L. The mass concentration of Lp(a) is denoted in mg/dL while the molar concentration is in nmol/L [6]. Notwithstanding, these concentrations are not equal or interchangeable, so their data cannot be compared between various studies. Consequently, a specific cutoff value for elevated Lp(a) and risk cannot be determined. Nevertheless, we can extrapolate that a value ≥50 mg/dL or ≥125 nmol/L confers enhanced risk similar to CAD as reported in the 2019 American College of Cardiology/American Heart Association guidelines on the primary prevention of cardiovascular disease [3].

The clinical studies have varied between retrospective, prospective, and meta-analysis, which showed that varying levels of elevated Lp(a) can induce PAD or worsen PAD outcomes. Given the extensive heterogeneity of the data, precise decisions about the benefits of isolated Lp(a) reduction cannot be surmised. Furthermore, some studies had small sample sizes with limited follow-up. Nevertheless, the aggregate data suggests that elevated Lp(a) causes a greater risk for PAD.

However, it remains unknown if lowering Lp(a) will significantly lower morbidity and mortality in PAD. Unifying Lp(a) measurements will be helpful so that future studies can be compared with similar measurement values. Moreover, determining a specific threshold at which patients have an increased risk for PAD is worthwhile. Furthermore, once patients develop CAD or PAD, it remains unknown if there is a specific value we should target for reduction.

Since Lp(a) is primarily genetically predetermined, repeated Lp(a) measurements are unnecessary. A single measurement can serve as a baseline value or aid in determining if an individual has a risk factor for CAD or PAD. Therefore, understanding the importance of Lp(a) as a risk factor for not only CAD, but also PAD can provide insight into PAD management. Lp(a) can potentially add prognostic value in patients who may have CAD to understand if they are at an increased risk of PAD. For example, a patient with CAD and elevated Lp(a) may have PAD or polyvascular disease. Thus, patients who have IM may be considered for more intensive lipid management and exercise therapy for PAD.

Another example is that if a patient has symptoms of IM with an ultrasound or ankle brachial index revealing PAD, Lp(a) may be measured for risk stratification. If Lp(a) is high, counseling may be provided about aggressive lifestyle modifications, statin therapy, aspirin consumption, smoking cessation and a “heart healthy” diet to minimize the progression of PAD. This may also mitigate the patient’s risk of subsequent CAD, MI, or stroke. In patients with severe PAD or a history of peripheral intervention, obtaining a Lp(a) measurement can determine if Lp(a) apheresis is a possible therapeutic option.

While all ethnicities are at risk of having an elevated Lp(a), African Americans have the highest Lp(a) levels [11]. Next are South Asians, meaning careful monitoring of their cardiovascular and PAD risk profile is critical to mitigate their comorbidities. In comparison, Caucasians, Hispanics and East Asians have lower levels [11]. In the Multi-Ethnic Study of Atherosclerosis (MESA) study, African Americans had the highest Lp(a) levels; however, this did not translate into higher incidence of PAD [36]. Interestingly, Hispanic men and women had a greater association of elevated Lp(a) with PAD even though African Americans had a higher level [36]. Nevertheless, there is insufficient data to expand upon specific ethnicity profiles for PAD. PAD and Lp(a) are not as extensively studied as in coronary disease. Moreover, South Asians were not evaluated in the MESA study [36]. South Asian ancestry is a risk factor noted in the AHA/ACC guideline for CAD [3].

Since Lp(a) can increase the risk of PAD, screening for Lp(a) can be considered in high-risk patients. Patients with FH, severely elevated LDL, early onset PAD, early onset CAD, severe CAD or PAD, multivessel CAD, PAD with multiple prior revascularizations, and multiple strokes are possible candidates for Lp(a) screening. Since South Asians are a high-risk group, they can be screened if they have CAD or PAD. In patients with both severe atherosclerotic disease and elevated Lp(a), aggressive lipid management should be implemented to mitigate their risk of worsening cardiovascular outcomes. Moreover, improved control of their other cardiac risk factors such as hypertension, diabetes, chronic kidney disease, LDL, metabolic syndrome, and obesity should be considered. Optimizing and maximizing medical therapy should be implemented in such patients to curtail the negative impacts of elevated Lp(a). Whether the isolated treatment of elevated Lp(a) can have a clinically meaningful impact for PAD remains unknown suggesting an area of further research.

Once the ongoing clinical trials of siRNAs and ASOs are completed, we may have targeted therapies for an elevated Lp(a). Such therapies may provide an enormous benefit to patients with CAD and PAD. In patients with optimally controlled LDL, triglycerides, and normal HDL who subsequently develop CAD, Lp(a) measurement can be considered. The mainstay of therapy should consist of LDL and cholesterol reduction, but Lp(a) serves as a risk-enhancing factor which can be measured once for risk stratification and counseling. In patients with simultaneous elevation of Lp(a) and LDL, the primary goal should be the reduction of LDL. Therefore, the compilation of data suggests a positive correlation between elevated Lp(a) and incidence of PAD or worse outcomes in pre-existing PAD, which is independent of other cardiac or atherosclerotic risk factors.

### Limitations

The limitations of this review include the scant literature on the involvement of elevated Lp(a) in PAD. Also, the clinical studies evaluating Lp(a) in PAD have not included randomized controlled trials so they may have been biased by confounding factors. Moreover, clinical studies had various values for Lp(a), hindering the generalizability of the results. Lastly, the use of nmol/L or mg/dL in Lp(a) measurement complicates the assessment of specific values to define elevated Lp(a) and the interpretation of the data.

## 8. Conclusions

Elevated Lp(a) is associated with an increased risk for PAD, CAD, MI, and stroke. Elevated Lp(a) acts an additional risk factor for atherosclerosis in the cardiovascular system, which can induce polyvascular disease. It can contribute to PAD and increase the risk of repeat revascularization after peripheral endovascular intervention. Patients with polyvascular disease have an enhanced risk for worse cardiovascular outcomes. Testing for Lp(a) should be considered in patients with CAD or PAD who have low risk factors or adequately controlled cholesterol levels. Currently no targeted FDA-approved medical therapy exists for lowering Lp(a). However, alirocumab, evolocumab, and inclisiran can reduce LDL and Lp(a) levels. Lp(a) apheresis is the only FDAapproved method for reduction of Lp(a) from the blood. Clinical trials including siRNA and ASO are currently underway for the targeted treatment of Lp(a) reduction.

## Availability of Data and Materials

All data generated or analyzed during this study are included in this published article.
